# Ecotypic differentiation and phenotypic plasticity combine to enhance the invasiveness of the most widespread daisy in Chile, *Leontodon saxatilis*

**DOI:** 10.1038/s41598-017-01457-1

**Published:** 2017-05-08

**Authors:** Irene Martín-Forés, Marta Avilés, Belén Acosta-Gallo, Martin F. Breed, Alejandro del Pozo, José M. de Miguel, Laura Sánchez-Jardón, Isabel Castro, Carlos Ovalle, Miguel A. Casado

**Affiliations:** 1 Complutense University of Madrid, Department of Ecology, Madrid, Spain; 2 King Juan Carlos University, Móstoles, Madrid, Spain; 3 School of Biological Sciences, The University of Adelaide, Adelaide, Australia; 4 University of Talca, Faculty of Agricultural Sciences, Talca, Chile; 5 Autonomous University of Madrid, Department of Ecology, Madrid, Spain; 6 Agricultural Research Institute INIA-La Cruz, La Cruz, Chile

## Abstract

Dispersal and reproductive traits of successful plant invaders are expected to undergo strong selection during biological invasions. Numerous Asteraceae are invasive and display dimorphic fruits within a single flower head, resulting in differential dispersal pathways - wind-dispersed fruits vs. non-dispersing fruits. We explored ecotypic differentiation and phenotypic plasticity of seed output and fruit dimorphisms in exotic Chilean and native Spanish populations of *Leontodon saxatilis* subsp. *rothii*. We collected flower heads from populations in Spain and Chile along a rainfall gradient. Seeds from all populations were planted in reciprocal transplant trials in Spain and Chile to explore their performance in the native and invasive range. We scored plant biomass, reproductive investment and fruit dimorphism. We observed strong plasticity, where plants grown in the invasive range had much greater biomass, flower head size and seed output, with a higher proportion of wind-dispersed fruits, than those grown in the native range. We also observed a significant ecotype effect, where the exotic populations displayed higher proportions of wind-dispersed fruits than native populations. Together, these patterns reflect a combination of phenotypic plasticity and ecotypic differentiation, indicating that *Leontodon saxatilis* has probably increased propagule pressure and dispersal distances in its invasive range to enhance its invasiveness.

## Introduction

The impacts of invasive plant species on resident communities and ecosystem functions are a global concern, which has led to considerable resources being invested into studying invasiveness. Of particular importance is predicting which plants will become invasive. The invasiveness of alien plants depends on the habitat characteristics of the recipient area (e.g. the fluctuating resource availability theory^1^), as well as on species traits^2^. The characteristics of recipient habitats have received considerable attention^3, 4^, highlighting that the habitats more prone to be invaded are those that are more productive or more disturbed^5, 6^. However, despite some recent attention, understanding the role played by species traits in the invasion process still remains a key knowledge gap in invasion biology^7, 8^.

These knowledge gaps exist because invasion success is primarily studied species-by-species^9, 10^, but these gaps also exist because most studies do not compare species traits in native and invasive ranges^11^. Nevertheless, some plant traits related to reproductive and dispersal characteristics have been suggested to be of key importance to invasiveness, such as plant growth rate, seed size, and distance of seed dispersal^7, 12–14^. For example, previous studies have shown that greater plant growth accounts for the invasiveness of many alien plant species^15^. Likewise, there is a correlation between seed size and greater invasiveness, where species with numerous smaller seeds have higher abundances and numbers of sites occupied than species with larger seeds^7, 16^.

Distance of seed dispersal appears to be positively related with both spread rate and geographic range size of invasive plants^17^. In order to successfully colonize new environments, plants largely rely on two main strategies: enhancing seed output and/or increasing the distance of seed dispersal^18, 19^. Greater seed output is determined by the fraction of total biomass allocated to reproduction^20, 21^. Increasing the distance of seed dispersal results from selection acting on the dispersal traits or due to maternal effects^22, 23^.

The dispersal syndrome of a plant can be inferred from the morphology of its propagules^24^. Plants use polymorphic seeds and fruits to overcome different microhabitats and environmental conditions^25^, allowing the colonization of highly unpredictable habitats^26, 27^. Therefore, it can be expected that seed output and distance of seed dispersal are likely to undergo strong positive selection during plant invasions^28, 29^, although more studies are needed to elucidate the roles of plasticity and selection acting on these traits^30, 31^.

Many invasive plant species are Asteraceae^32^, and interestingly, many Asteraceae also present fruit (i.e. achene) dimorphisms (i.e. heterocarpy^26^). *Leontodon saxatilis* subsp. *rothii* Maire is a daisy common in the Mediterranean Basin. It is native and typical of Spanish grasslands, although it is now widely naturalized in Chile, plus other Mediterranean regions such as California^33^ and southern Australia^34^. *Leontodon saxatilis* constitutes an ideal candidate to study the importance and evolution of dispersal traits during invasions because it produces two morphologically distinct fruits and it has recently undergone a successful invasion. It produces peripheral fruits that lack dispersal structures, plus central fruits that are dispersed by wind.

The aim of this study was to explore plasticity and ecotypic differences in seed output and fruit dimorphism patterns associated with the colonization of *L*. *saxatilis* into Chile. We collected *L*. *saxatilis* fruits from 5 populations in its native range in Spain and 4 populations in its invasive range in Chile. We grew these plants in common garden trials at both its origin and its invasive range, and quantified the ecotypic and plastic components of variation in reproductive and dispersal-related traits. An ecotype effect would be observed where the geographic origin of plants explains differences in reproductive and dispersal-related traits at the same common garden trial. Similarly, phenotypic plasticity would be observed where individuals from the same geographic origin produce different reproductive and dispersal-related trait values in the two common garden trials. We expected that plants grown in the invasive range would be more productive because of reduced competition and enemy release. Similarly, we expected that plants from the invasive range would be more productive because, during invasion, it is likely that selection occurred on traits that allowed plants to undergo longer distance seed dispersal and produce greater amounts of seed.

## Results

We use the term biomass to refer to above-ground biomass, number of fruits per flower head to refer to average number of fruits per flower head, seed output per plant to refer to total estimated seed output per plant, and proportion of central wind dispersed fruits to refer to average proportion of central fruits within a flower head. Likewise, we use the simplified names of the variables even though some of them were transformed to carry out the analyses, with details of transformations in the methods. In the case of predictor variables, Site represents the country of reciprocal transplant trials (Chile or Spain), whereas Origin corresponds to the country of source populations (Chile or Spain). According to the linear models, both Site and Origin explained most of the variation of our response variables.

### Plant growth

Planting site had the largest effect on biomass, with larger plants in Chile (mean biomass in Chile = 25.8 g ± 1.19 SE; in Spain = 6.7 g ± 0.3 SE; P < 0.001; Table 1; see Supplementary Table S1 online for detailed information). To a lesser extent, biomass was also influenced by precipitation of the source population. The site*precipitation effect was also significant. For populations grown in Chile, there was a trend of larger biomass for populations from lower rainfall areas (r = 0.361; P < 0.001), whereas for populations grown in Spain, precipitation and biomass were not correlated (r = 0.036; P > 0.05).

**Table 1:** Model coefficients (and t/z values) for the selection of linear models after applying the parsimony criterion on the subset of best models based on AICc, regarding the effects of the common garden trial site (Site), country of origin (Origin), and annual precipitation of source populations (Precip) on Leontodon taraxacoides phenological traits: biomass (Biomass), number of flower heads (NFlowerHeads), number of fruits per flower head (FruitsFH), seed output (SeedOutput), and the proportion of central fruits (PCF).

	Biomass	NFlowerHeads	FruitsFH	SeedOutput	PCF
Intercept	1.37	4.20	150.40	3.96	0.89
	(41.8***)	(49.6***)	(33.2***)	(100.11***)	(85.2***)
Site	−0.59	−0.42	−55.33	−0.46	−0.03
	(−14.0***)	(−3.8***)	(−11.6***)	(−9.3**)	(−3.5***)
Origin		0.22			−0.06
		(1.8*)			(−4.3***)
Precip	−0.10				
	(−2.9**)				
Site*Origin		−0.43			
		(−2.8***)			
Site*Precip	0.11				
	(2.4**)				

Source population and subplot nested within site was considered as random factors in every model. All models were fitted to Gaussian distribution except for NFlowerHeads where a Poisson function was used. Biomass and SeedOutput were log-transformed for linearity prior to analyses.

Significance codes: *** ≤ 0.001, ** ≤ 0.01; * ≤ 0.05.

### Reproductive investment

Variation in all three fitness traits related to reproductive investment (i.e. number of flower heads per plant, number of fruits per flower head, and seed output per plant) was largely explained by planting site, being greater for the three variables in the Chilean common garden than in the Spanish one. The country of origin of the populations also had a significant but weak effect on the number of flower heads per plant and the number of fruits per flower head with higher values for the populations coming from Chile.

**Figure 1:**
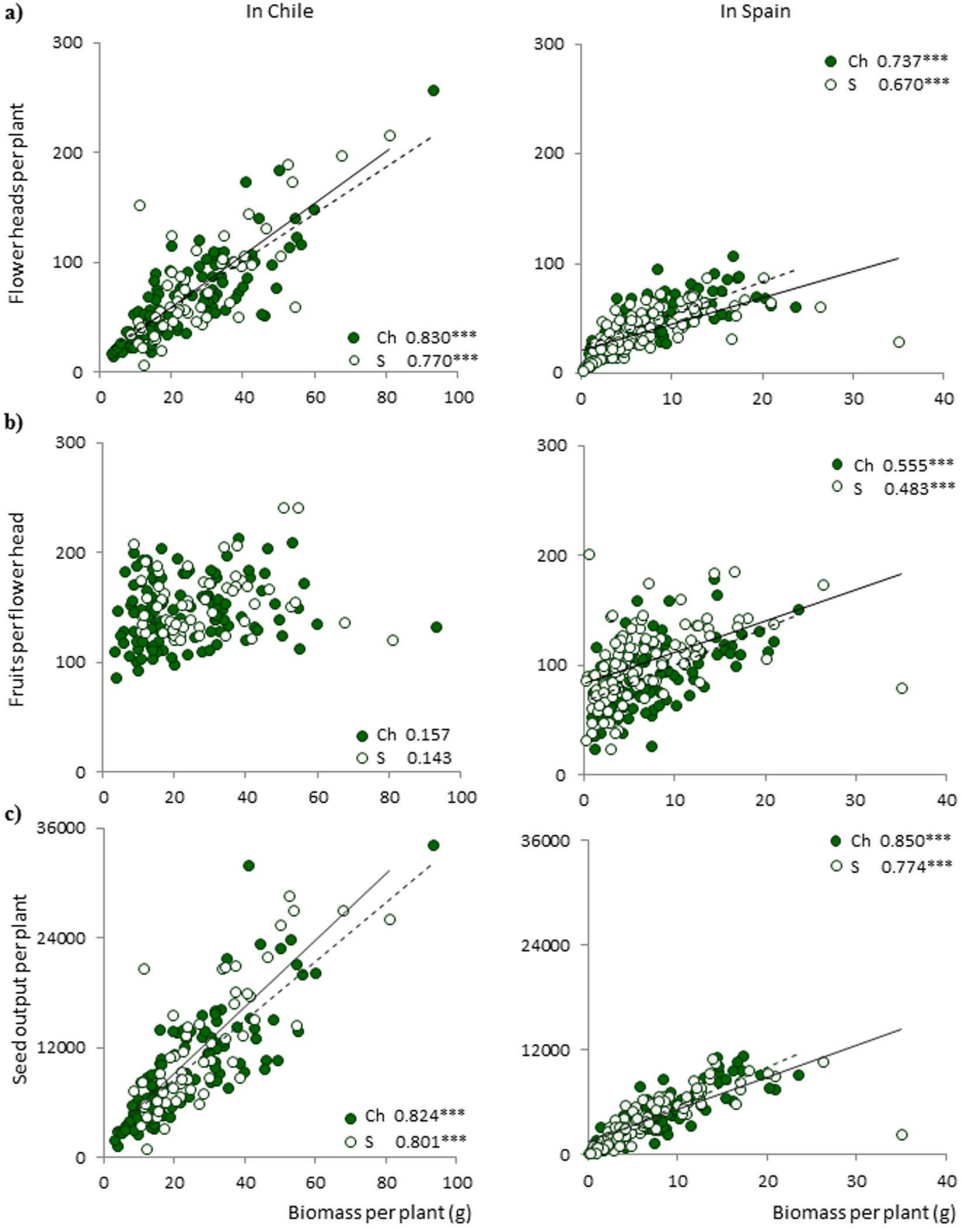
Relationships between biomass and (**a**) number of flower heads per plant, (**b**) number of fruits per flower head and (**c**) seed output per plant at each site. Close circles represent Chilean populations (Ch) whereas open ones refer to Spanish ones (S). Significant relationships are shown by discontinuous (Chilean populations) or continuous (Spanish populations) lines. For each relationship, regression coefficient and its significance are shown (* < 0.05; ** < 0.01; *** < 0.001).

All the variables reflecting reproductive investment were significantly and positively correlated with biomass (Fig. 1a–c), regardless of the planting site and the origin of the population (number of flower heads per plant: r^2^ = 0.7–0.8; number of fruits per flower head: r^2^ = 0.5–0.6 but only for the common garden in Spain; seed output: r^2^ = 0.8–0.9). The regression slopes between the number of fruits per flower head and biomass were ten times steeper in Spain than in Chile, regardless of the country of origin of populations (3.37 and 2.84 for Chilean and Spanish populations grown in Spain, and 0.30 and 0.27 for Chilean and Spanish populations grown in Chile, respectively).

### Dispersal strategy

**Figure 2:**
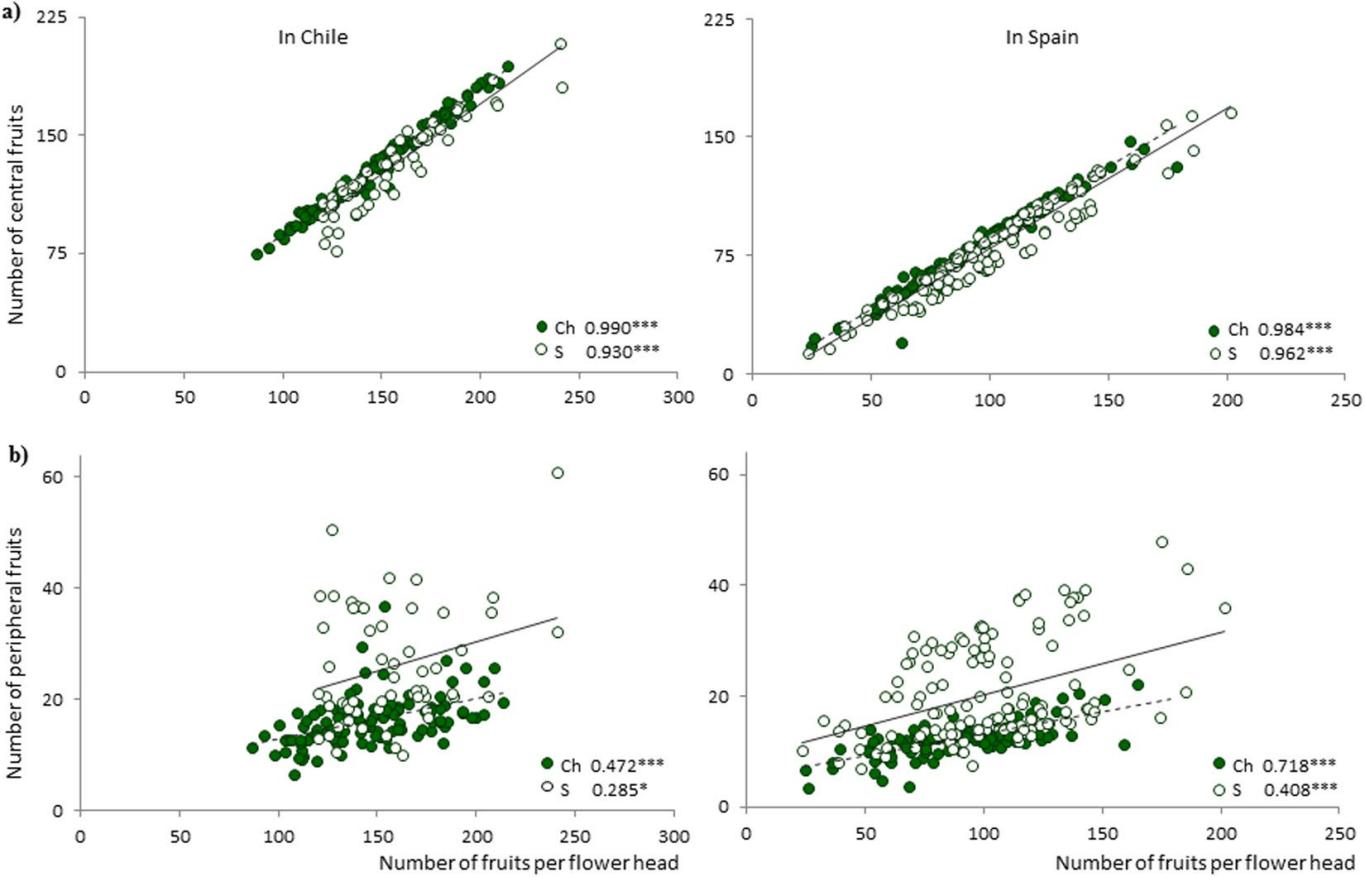
Relationships between (**a**) number of central wind-dispersed and (**b**) peripheral non-dispersing fruits and the number of fruits per flower head at each site. Close circles represent Chilean populations (Ch) whereas open ones refer to Spanish ones (S). Significant relationships are shown by discontinuous (Chilean populations) or continuous (Spanish populations) lines. For each relationship, regression coefficient and its significance are shown (* < 0.05; ** < 0.01; *** < 0.001).

Both central (wind-dispersed) and peripheral (non-dispersing) fruits increased with the number of fruits per flower head in populations from both countries grown in the native and the invaded ranges, although this trend was especially noticeable for central fruits (central fruit regression coefficients r^2^ > 0.9 in both sites; peripheral fruit regression coefficients r^2^ 0.2 to 0.5 in Chile and r^2^ 0.4 to 0.7 in Spain) (Fig. 2a,b).

The proportion of central fruits was significantly higher in the Chilean common garden, although the country of origin of the populations also explained this variable (Table 1). Chilean populations always had a higher proportion of central fruits than Spanish populations (mean proportion of central fruits: in Chile = 0.87 ± 0.004 SE, in Spain = 0.81 ± 0.005 SE; mean proportion of central fruits: for Chilean populations = 0.87 ± 0.003 SE, for Spanish populations = 0.80 ± 0.006 SE).

## Discussion

The importance of comparing invasive plant performance in reciprocal transplant trials across native and invasive ranges has only recently been emphasized^35^. Our study represents one of the first to track key invasive traits via reciprocal transplant trials established in both the native and invasive range of a widely distributed and impactful invasive species^36–38^. We showed that the most common alien plant species in Chile, the daisy *Leontodon saxatilis*, combined multiple strategies in being a successful invader from its native Spanish range into Chile (a combination that is consistent with that observed for other species^39^). It displayed strong phenotypic plasticity in reproductive investment (i.e. number of flower heads produced, number of fruits per flower head, and seed output) and in the proportion of central wind-dispersed fruits (i.e. the reproductive investment and the proportion of wind-dispersed fruits), which were both greater in the invasive range than in the native one. This species also displayed strong ecotypic differentiation for the proportion of central wind-dispersed fruits, and significant but weaker ecotypic effects for the number of flower heads per plant and the number of fruits per flower head. These trends suggest that *L*. *saxatilis* has undergone strong selection for bigger flower heads and longer distance seed dispersal. In addition, *L*. *saxatilis* displayed greater growth potential in the novel environment, possibly as a result of fewer competitors (see examples of enemy release^40^). In combination, these traits should increase the invasiveness of *L*. *saxatilis* by increasing propagule pressure and the distance of seed dispersal in its invasive range.

The residence time hypothesis proposes that the longer an alien species is present in a new environment, the higher the chances that it will become a successful colonizer^41, 42^. The first record of *L*. *saxatilis* in Chile was 1963^43^. Due to its short residence time, a narrow regional distribution of this species in Chile would be expected^43–45^. However, since its arrival, it has become widely distributed (from 32°S to 42°S) and is present in several administrative regions far beyond the Mediterranean area^46^, indicating an unexpectedly high invasiveness. *L* *eontodon* *saxatilis* is the most frequent alien species in Chile^47^, which provides further evidence of its success in colonizing new areas and overcoming a diversity of novel conditions^48^. Anthropogenic activities seem to determine the spatial distribution of *L*. *saxatilis* in its invasive range^49^. The colonization ability of this species depends mostly on its capacity to colonize areas created after human disturbance (in line with the ‘novel niche hypothesis’^50^) and its great dispersal ability (in line with the ‘propagule pressure hypothesis’^51^).

Once *L*. *saxatilis* arrives into an exotic location it most likely follows a combined bet-hedging strategy^52, 53^. Central wind-dispersed fruits are always higher in number and spread further than peripheral fruits. However, wind-dispersal is a high-risk strategy (i.e. wind-dispersed fruits have a high probability of falling in unfavourable sites), and is strongly affected by stochastic events. The peripheral fruits lack dispersal structures and rely on a low risk strategy. Peripheral fruits are larger, with a thicker pericarp and are viable for longer than central fruits^54, 55^. Peripheral fruits rely on a self-replacement strategy^56^, being most able to colonize the location where their mother plant grew^57^. The low risk, self-replacement strategy increases the chances of peripheral fruits supporting the naturalization of *L*. *saxatilis* in the vicinity of the mother plant, which allows it to overcome the challenges of the ‘novel niche hypothesis’^58^. Conversely, the high risk strategy allows wind-dispersed fruits to achieve longer distance seed dispersal and thus reach areas further afield from mother plants^57, 59^. This longer distance of seed dispersal would help expand the distribution of this species and overcome the challenges related to the ‘propagule pressure hypothesis’.

We demonstrated that the plant growth and reproductive investment of *L*. *saxatilis* had strong phenotypic plasticity and, to a lesser extent, ecotypic differentiation. *Leontodon saxatilis* biomass was far greater when it grew in its invasive range than when it grew in its native range, which is similar to other alien species^60–63^.

Biomass produced by this species was invested in maximizing reproduction, enhancing the two fitness parameters involved in seed output – the number of flower heads produced per plant, and the number of fruits produced per flower head. Seed output displayed by *L*. *saxatilis* was highly dependent on biomass, which also showed strong phenotypic plasticity. Seed output was significantly greater for individuals grown in their invasive range, constituting an effective strategy for this species to produce more dispersal units and promote its propagule pressure in a novel environment.

The number of fruits produced by *L*. *saxatilis* affects its dispersal ability. We showed that fitness increased with biomass, however the number of fruits produced per flower head had an asymptotic response as a consequence of an upper limit in the size that the flower head could reach. The number of fruits per flower head in the invasive range was greater than in the native range, however the relationship between the number of fruits per flower head and biomass was significant but non-linear. The weak linear association between the number of fruits per flower head and biomass indicates that the flower head has architectural constraints. In Asteraceae flower head size is restricted by the receptacle (i.e. the surface of the flower head where the fruits are implanted) and can only increase up to a limit that characterizes each species.

We showed that all plants in our study increased the number of central wind-dispersed and peripheral non-dispersing fruits with the number of fruits per flower head. However, this relationship was stronger for central fruits than for peripheral fruits (central fruits: r^2^ > 0.9; peripheral fruits r^2^ = 0.2–0.7), and the regression slope between central fruits and biomass was an order of magnitude greater than the regression slope for peripheral fruits and biomass. Considering that the number of fruits per flower head is a surrogate for the size of flower heads (i.e. it is limited by the surface of the receptacle), the different performance of the two types of fruits is again likely to be due to architectural constrictions. When the radius of the receptacle increases, the number of peripheral fruits (arranged in one row at the edge in the involucral bracts) increases with the length of the circumference, whereas the number of central fruits increases with the surface area of the receptacle. Therefore, the carrying capacity of the flower head differs for each type of fruit. As the number of fruits per flower head was significantly higher for populations grown in the exotic site, more central wind-dispersed fruits were produced in the invasive range.

The proportion of wind-dispersed fruits produced within a flower head also presented greater phenotypic plasticity for individuals grown in the invasive range. Nevertheless, the proportion of wind-dispersed fruits mostly presented ecotypic differentiation, where variation in the proportion of wind-dispersed fruits was best explained by the country of origin of populations. The proportion of wind-dispersed fruits was significantly greater in Chilean populations than in Spanish ones, which suggests that selection for longer-dispersal ability had occurred in Chile. Ecotypic differentiation has also been reported for other Asteraceae, such as *Crepis sancta* ^64^. Having a greater proportion of wind-dispersed fruits results in maximizing seed dispersal in the colonized area, thus the invasiveness of *L*. *saxatilis* appears to be greater for exotic populations and for individuals grown in its invasive range.

In summary, we show that colonization ability is an important component of a plant’s invasiveness. Some invasive plant species, such as the daisy *L*. *saxatilis* studied here, appear to combine phenotypic plasticity and ecotypic differentiation as coadaptations to cope with the novel conditions of the invasive range and to increase propagule pressure and distance of seed dispersal in its new environment. *Leontodon saxatilis*, the most frequently observed species in central Chile, seems to only require simple mechanisms to increase its capacity of invasive expansion. A combination of simple architectural relationships, largely dependent on biomass (i.e. the number and size of flower heads), and different dispersal strategies (i.e. wind vs. non-dispersing) impact on the invasiveness of *L*. *saxatilis* in its invasive range. Alien species with fruit dimorphisms should be carefully controlled and their spread monitored, with daisies requiring special attention due to their great invasive potential. Consequently, studying the evolution of dispersal abilities of invasive species is important for understanding invasiveness and therefore management of biological invasions and conservation policies.

## Methods

### Study species

A large proportion of species from the Mediterranean Basin were introduced to Chile during the Spanish conquest of Latin America and many of these species have become naturalized in the Mediterranean climatic region of central Chile. The percentage of invasive species in central Chile that belong to the Asteraceae family is ca. 13.9%^47^. Most daisies produce two types of fruits that occupy different locations on the flower head. Fruits are either on the periphery or in the center, and they show different morphologies, germination requirements and dispersal abilities^65, 66^. Central fruits are generally smaller, lighter and have structures that allow wind-dispersal (anemochory). Peripheral fruits are larger, heavier and lack specific structures for wind-dispersal^67, 68^, but often have modifications that make them suitable for animal (passive-zoochory^66^) or water dispersal (hydrochory^67^).

*Leontodon saxatilis* subsp. *rothii* produces dark brown heavy fruits without a pappus or other specific dispersal structures and light brown fruits with a pappus that are wind-dispersed. *Leontodon saxatilis* subsp. *rothii* is the accepted name of the species although it has many recognized synonyms (e.g. *Colobium hispidum* Roth, *Leontodon longirostris* (Finch & P.D. Sell) Talavera, *Leontodon saxatilis* subsp. *hispidus* (Roth) Castrov. & M. Laínz, *Leontodon saxatilis* subsp. *longirostris* (Finch & P.D. Sell) P. Silva, *Leontodon taraxacoides* subsp. *hispidus* (Roth) Kerguélen, *Leontodon taraxacoides* subsp. *longirostris* Finch & P.D. Sell, *Thrincia hispida* (Roth) Roth, *Thrincia saxatilis* subsp. *hispida* (Roth) Holub & Moravec^69, 70^).

### Study area

**Figure 3:**
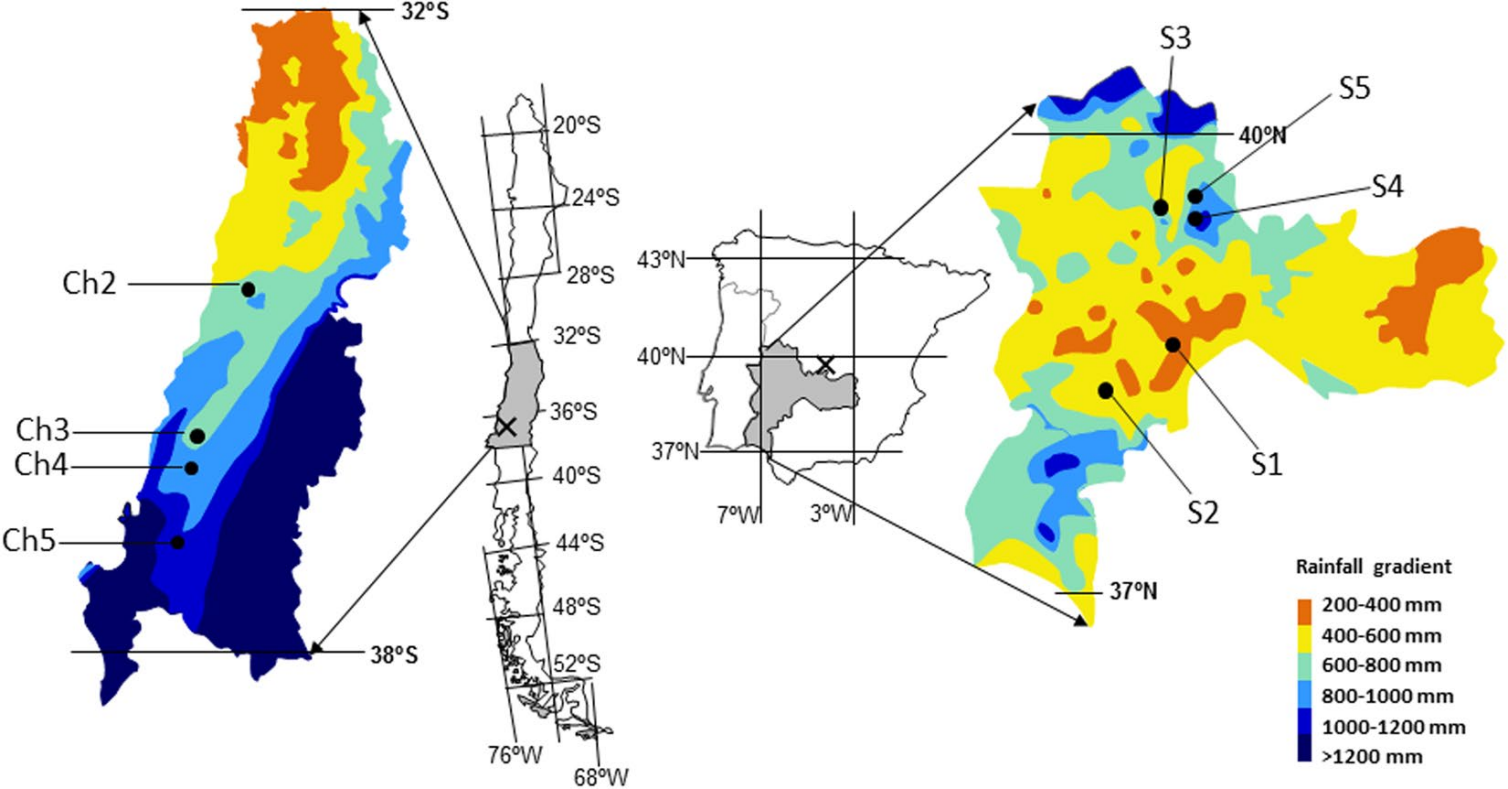
Map of the studied areas of Mediterranean grasslands in Spain and Chile, including sampling sites (see Table 2). Grey tones represent rainfall variability in each country. The locations of the reciprocal transplant trials are shown (x). The figure and the maps were created manually using Microsoft PowerPoint version 14.0.7166.5000 by modifying images from Google Maps (Microsoft Office Professional Plus 2010; https://microsoft-office-professional-plus.uptodown.com/windows; map of Chile (https://www.google.es/maps/place/Chile/@-35.3617722,-89.1181162,4z/data=!3m1!4b1!4m5!3m4!1s0×9662c5410425af2f:0×505e1131102b91d!8m2!3d-35.675147!4d-71.542969 (Map Data ©2016 Google, INEGI)); map of Spain (https://www.google.es/maps/place/Espa%C3%B1a/@40.1300278,-8.2052927,6z/data=!3m1!4b1!4m5!3m4!1s0xc42e3783261bc8b:0xa6ec2c940768a3ec!8m2!3d40.463667!4d-3.74922 (Map Data ©2016 Google)).

Our study was based in the Mediterranean grasslands of Chile and Spain. In Chile, populations of *L*. *saxatilis* were collected in the central region (from 32°31′ to 37°00′ S and 70°46′ to 72°34′ W), with mean annual precipitation between 300 and 1200 mm. In Spain, the populations were collected in the center-west of the Iberian Peninsula (from 37°51′ to 40°14′N and from 4°23′ to 7°02′W), with mean annual precipitation between 400 and 1100 mm (Fig. 3). Both regions are similar in terms of lithology (acid substrate, derived from igneous or metamorphic rocks), plant physiognomy (the structure consists of a continuous herbaceous layer with scattered trees), and land use (mainly extensive livestock grazing by sheep and cattle). Almost half of the species present in the Mediterranean Chilean grasslands are aliens that largely originated in the Mediterranean Basin^47^, and the Iberian Peninsula^71^.

### Data collection

We selected populations of *L*. *saxatilis* representative of the rainfall gradient existing in the Mediterranean regions of Spain and Chile. Five populations were sampled in Spain, and four in Chile (Fig. 3; Table 2). We collected mature flower heads from 50 individuals of *L*. *saxatilis* for each population in spring of 2010 (May and June in Spanish sites, and October and November in Chilean sites). Geographic coordinates, altitude and climate conditions including annual precipitation and mean annual temperature were recorded at each site (Table 2). Climate data were obtained from the State Meteorological Agency (AEMET, http://www.aemet.es) and the *Atlas Climático Digital de la Península Ibérica* ^72^ for Spain, and from WorldClim^73^ for Chile.

**Table 2:** Geographic and climatic characteristics of the studied populations.

Country	Pop	Site	Latitude	Longitude	Temp (°C)	Precip (mm)
Chile	Ch2	Pumanque	34°37′48″S	71°42′54″W	15.01	719
	Ch3	Boldo	35°58′52″S	72°13'38″W	14.33	794
	Ch4	Quirihue	36°15′20″S	72°32′58″W	13.14	972
	Ch5	Yumbel	37°00′26″S	72°34′01″W	13.33	1168
Spain	S1	Castuera	38°46′20″N	5°34′48″W	16.89	468
	S2	Fuente de Canto	38°16′33″N	6°20′22″W	15.81	572
	S3	Madroñera	39°25′23″N	5°47′48″W	15.42	666
	S4	Ibor	39°32′53″N	5°22′57″W	14.46	859
	S5	Logrosán	39°21′28″N	5°25′04″W	16.17	913

Pop = population code. Temp = mean annual temperature. Precip = mean total annual precipitation.

Flower heads were cleaned in the lab to obtain fruits. Peripheral fruits were chosen for subsequent planting because of their greater success in pre-germination studies (see previous studies^57, 68^). Seeds from each population were germinated in petri dishes and then transplanted into subplots within two common garden trials (hereafter sites). Sites were the Experimental Centre of Cauquenes-INIA, Chile (35°58′ S, 72°17′ W; 140 m a.s.l.; 14.4 °C; 748 mm of mean annual precipitation) and the Faculty of Agronomy of the Polytechnic University of Madrid, Spain (40°26′N, 3°44′W; 600 m a.s.l.; 15 °C of mean annual temperature; 484 mm. of mean annual precipitation). The conditions within both common gardens were controlled so there was no herbivory or competition that could affect the experiment results. Ideally, including replicates of common garden experiments within each country would have been desirable to explore the effect of site^74^, but in this case it was not possible due to logistical and bioethical restrictions.

Planting was conducted in June 2012 in Chile and October 2012 in Spain, and sites were prepared by removing surface vegetation. Ten and 20 seedlings of each population were planted in subplots of 100 × 50 cm long and 200 × 50 cm long in Chile and Spain, respectively. The distance between plants was 20 cm and the separation between subplots was 30 cm. A complete randomized design was used with three replicate subplots. Thus, in each common garden there were a total of 27 subplots: 15 containing populations from Spain (5 populations * 3 replicates), and 12 containing populations from Chile (4 populations * 3 replicates). The total number of individuals planted was 270 in Chile and 540 in Spain. During the period of the experiment, the amount of rainfall was similar in both sites, although slightly higher in Chile (218.3 mm in Spain and 284.8 mm in Chile). However, the temporal distribution of rainfall during this period differed for both countries, with Spain being drier in winter and more humid in spring than Chile. Throughout the experiment, 39% and 55% of individuals died in Chile and Spain, respectively, possibly influenced by these weather conditions. The final count of survivors included in our study was 166 in Chile and 245 in Spain. The fact that the individuals arranged in the reciprocal transplant trials differed between countries was due to the availability of space to carry out the experiment in both sites, which was greater in Spain than in Chile. Flower heads were collected from every individual after they were mature but before the infructescence opened, to ensure we captured all seeds.

Seed output and proportion of central (wind-dispersed) fruits were the target traits of our study because of their key role on invasiveness of *L*. *saxatilis*. When plants reached ca. 50% senescence (i.e. we observed that half of the flower heads had maturated), five flower heads were collected from each individual and traits associated with fitness and dispersal ability estimated. Plants were harvested when they reached 75% senescence.

Once all plants had been harvested, we measured aboveground biomass (hereafter biomass) and counted the number of flower heads per plant. Flower head size was measured by counting the number of fruits per flower head present in five flower heads; the central fruits were separated from the peripheral non-dispersing fruits. Then the average number of fruits per flower head was calculated. The average proportion of central fruits within a flower head was also calculated by dividing the average number of central fruits within a flower head by the average number of fruits per flower head. Finally, we estimated the total seed output per plant (i.e. the total number of seeds produced per individual) by multiplying the number of flower heads per plant by the average number of fruits per flower head.

### Data analyses

We used mixed effects models to analyze the colonization ability of *L*. *saxatilis*, considering the plant individual as the unit of analysis (n = 408). Models were fitted to the following response variables: plant growth (i.e. biomass), reproductive investment (number of flower heads per plant, average number of fruits per flower head and estimated seed output per plant), and the dispersal strategy (average proportion of central wind-dispersed fruits within a flower head). Predictor variables included the site of the reciprocal transplant trials (Chile or Spain), the country of origin of the source populations (Chile or Spain), and precipitation of the source populations. The source population and the subplot where populations were planted within each common garden (subplot nested within site) were included as random effects. All the possible models including site, origin and precipitation as predictors were computed.

We used mixed effects models with a Gaussian error distribution except for the number of flower heads per plant, where we used a Poisson link function. Biomass and estimated seed output per plant were log-transformed, and precipitation was rescaled by standardization to improve model fitting. Models computed for the proportion of central fruits were weighted by the number of fruits per flower head.

We compared the possible models differing in the structure of fixed effects fit by maximum likelihood whether they had a Gaussian error distribution and the Laplace approximation when they had a Poisson error distribution. We calculated the Akaike Information Criterion corrected for small sample size (AICc)^75^ and selected the best-fit models (= all models with ∆AICc < 2 from the best fitting model with the lowest AICc) (See Supplementary Table S1 online for detailed information). The parsimony criterion was then applied on the subset of best models based on AICc, where the model with the lowest number of parameters was chosen for subsequent analysis. Selected models were fitted by Restricted Maximum Likelihood. Model validation of the best-fit model was based on visually assessing the normality of residuals, and we tested for model overdispersion by checking that model residual deviance was lower than the residual degrees of freedom^76^.

We also ran regressions between biomass and the fitness traits (number of flower heads per plant, average number of fruits per flower head, and estimated seed output per plant), and between the size of the flower head (i.e. average number of fruits per flower head) and the number of fruits of each type, central and peripheral. All regressions were run by splitting the individual plants by site and origin into four groups: (i) native populations planted in the native range, (ii) exotic populations planted in the native range, (iii) native populations planted in the invaded range, and (iv) exotic populations planted in the invaded range.

All analyses were performed in R v 3.2.3^77^, using the base stats package plus the lme4^78^ and AICcmodavg^79^ packages. Outliers were defined as data that exceeded three times the interquartile range, and these were subsequently removed prior to analyses (=1.5% of cases).

## Electronic supplementary material

Supplementary Materials
