# Supplementary material for: Ecotypic differentiation and phenotypic plasticity combine to enhance the invasiveness of the most widespread daisy in Chile, Leontodon saxatilis
Source: Sci Rep. 2017 May 8;7:1546. doi: 10.1038/s41598-017-01457-1 (PMC5431524; doi:10.1038/s41598-017-01457-1)
Supplement: Supplementary file 1 — Supplementary Materials [file 41598_2017_1457_MOESM1_ESM.doc]

**Ecotypic differentiation and phenotypic plasticity combine to enhance the invasiveness of the most widespread daisy in Chile, *Leontodon saxatilis***

Irene Martín-Forés^1,2,3^*, Marta Avilés^1^, Belén Acosta-Gallo^1^, Martin F. Breed^3^, Alejandro del Pozo^4^, José M. de Miguel^1^, Laura Sánchez-Jardón^1^, Isabel Castro^5^, Carlos Ovalle^6^ & Miguel A. Casado^1^

^1^Complutense University of Madrid, Department of Ecology, Madrid, Spain

^2^King Juan Carlos University, Móstoles, Madrid, Spain

^3^School of Biological Sciences, The University of Adelaide, Australia

^4^University of Talca, Faculty of Agricultural Sciences, Talca, Chile

^5^Autonomous University of Madrid, Department of Ecology, Madrid, Spain

^6^Agricultural Research Institute INIA-La Cruz, La Cruz, Chile

*Author for correspondence: I. Martín-Forés ([imfores@pdi.ucm.es](mailto:imfores@pdi.ucm.es))

**Supplementary Table S1.** All the best models (lowest AICc and ∆AICc < 2) for each response variable. Selected models after applying parsimony criterion are highlighted in bold.

| **BIOMASS** | **Estimate** | **t value** |
| --- | --- | --- |
| **1.1) Biomass ~ Site * Precip + (1|Site:SubplotID) + (1|Pop)** | | |
| **(Intercept)** | **1.37128** | **41.76 ***** |
| **Site** | **-0.59436** | **-13.98 ***** |
| **Precip** | **-0.09967** | **-2.85 ***** |
| **Site * Precip** | **0.10542** | **2.44 ***** |
| 1.2) Biomass ~ Site * Precip + Origin + (1|Site:SubplotID) + (1|Pop) | |  |
| (Intercept) | 1.38351 | 32.48 *** |
| Site | -0.59346 | -13.94 *** |
| Precip | -0.10570 | -2.76 *** |
| Origin | -0.02680 | -0.48 |
| Site * Precip | 0.10461 | 2.41 *** |
| **NUMBER OF FLOWER HEADS** | **Estimate** | **z value** |
| 2.1) NFlowerHeads ~ Site * Origin * Precip + (1|Site:SubplotID) + (1|Pop) | | |
| (Intercept) | 4.24598 | 52.04 *** |
| Site | -0.51739 | -4.46 *** |
| Origin | 0.10102 | 0.87 |
| Precip | -0.09018 | -1.12 |
| Site * Origin | -0.36428 | -2.17 * |
| Site * Precip | 0.21745 | 1.90 . |
| Origin * Precip | -0.09331 | -0.72 |
| Site * Origin * Precip | -0.18493 | -1.08 |
| **2.2) NFlowerHeads ~ Site * Origin + (1|Site:SubplotID) + (1|Pop)** | |  |
| **(Intercept)** | **4.20431** | **49.60 ***** |
| **Site** | **-0.41720** | **-3.77 ***** |
| **Origin** | **0.21937** | **1.81 .** |
| **Site * Origin** | **-0.42948** | **-2.76 **** |
| 2.3) NFlowerHeads ~ Site * Origin + Precip + (1|Site:SubplotID) + (1|Pop) | | |
| (Intercept) | 4.23509 | 50.72 *** |
| Site | -0.41714 | -3.78 *** |
| Origin | 0.15825 | 1.30 |
| Precip | -0.06661 | -1.39 |
| Site * Origin | -0.44835 | -2.90 ** |
| 2.4) NFlowerHeads ~ Site * Precip + (1|Site:SubplotID) + (1|Pop) | |  |
| (Intercept) | 4.31953 | 71.66 *** |
| Site | -0.63749 | -8.13 *** |
| Precip | -0.15877 | -2.40 * |
| Site * Precip | 0.20126 | 2.49 * |
| **NUMBER OF FLOWER HEADS** | **Estimate** | **t value** |
| 3.1) FruitsFH ~ Site + Origin + (1|Site:SubplotID) + (1|Pop) | |  |
| (Intercept) | 145.076 | 26.97 *** |
| Site | -55.919 | -11.76 ** |
| Origin | 10.871 | 1.61 . |
| **3.2) FruitsFH ~ Site + (1|Site:SubplotID) + (1|Pop)** |  |  |
| **(Intercept)** | **150.404** | **33.21***** |
| **Site** | **-55.325** | **-11.61**** |
| 3.3) FruitsFH ~ Site + Precip + (1|Site:SubplotID) + (1|Pop) |  |  |
| (Intercept) | 150.386 | 34.38 *** |
| Site | -56.066 | -11.68 ** |
| Precip | -4.473 | -1.32 |
| 3.4) FruitsFH ~ Site * Origin + (1|Site:SubplotID) + (1|Pop) |  |  |
| (Intercept) | 146.858 | 24.97 *** |
| Site | -59.516 | -9.05 ** |
| Origin | 6.520 | 0.75 |
| Site * Origin | 7.695 | 0.80 |
| 3.5) FruitsFH ~ Site + Origin + Precip + (1|Site:SubplotID) + (1|Pop) | | |
| (Intercept) | 146.293 | 24.738*** |
| Site | -56.193 | -11.737 ** |
| Origin | 8.364 | 1.030 |
| Precip | -2.363 | -0.597 |
| **SEED OUTPUT** | **Estimate** | **t value** |
| **4.1) LogSeedOutput ~ Site + (1|Site:SubplotID) + (1|Pop)** |  |  |
| **(Intercept)** | **3.96187** | **100.11***** |
| **Site** | **-0.46463** | **-9.29 ***** |
| 4.2) LogSeedOutput ~ Site * Origin + Precip + (1|Site:SubplotID) + (1|Pop) | | |
| (Intercept) | 3.93588 | 71.38*** |
| Site | -0.38143 | -5.64 ** |
| Origin | 0.07390 | 0.87 |
| Precip | -0.04604 | -1.39 * |
| Site * Origin | -0.19386 | -1.96 ** |
| 4.3) LogSeedOutput ~ Site + Precip + (1|Site:SubplotID) + (1|Pop) | | |
| (Intercept) | 3.96507 | 101.55*** |
| Site | -0.47383 | -9.34 * |
| Precip | -0.03343 | -1.23 . |
| 4.4) LogSeedOutput ~ Site * Precip + (1|Site:SubplotID) + (1|Pop) | |  |
| (Intercept) | 3.97188 | 100.58*** |
| Site | -0.47552 | -9.50 *** |
| Precip | -0.07872 | -1.87 ** |
| Site * Precip | 0.07292 | 1.43 * |
| 4.5) LogSeedOutput ~ Site*Origin |  |  |
| (Intercept) | 3.91338 | 71.27 *** |
| Site | -0.37992 | -5.63 * |
| Origin | 0.11645 | 1.41 . |
| Site * Origin | -0.18302 | -1.86** |
| **PROPORTION OF WIND-DISPERSED FRUITS** | **Estimate** | **t value** |
| **5.1) PCF ~ Site + Origin + (1|Site:SubplotID) + (1|Pop)** |  |  |
| **(Intercept)** | **0.888620** | **85.22***** |
| **Site** | **-0.030093** | **-3.50 ***** |
| **Origin** | **-0.057748** | **-4.29 ***** |
| 5.2) PCF ~ Site + Origin + Precip + (1|Site:SubplotID) + (1|Pop) | |  |
| (Intercept) | 0.885691 | 75.90 *** |
| Site | -0.029449 | -3.41 *** |
| Origin | -0.051660 | -3.13 *** |
| Precip | 0.005718 | 0.71 |

Signif. codes: 0 ‘***’ 0.001 ‘**’ 0.01 ‘*’ 0.05 ‘.’ 0.1 ‘ ’ 1
